# Optimizing Outcomes of Postmastectomy Breast Reconstruction With Acellular Dermal Matrix: A Review of Recent Clinical Data

**Published:** 2017-06-12

**Authors:** Michael Zenn, Mark Venturi, Troy Pittman, Scott Spear, Geoffrey Gurtner, Geoffrey Robb, Alex Mesbahi, Joseph Dayan

**Affiliations:** ^a^CARE Plastic Surgery, Cary, NC; ^b^Department of Plastic Surgery, MedStar Georgetown University Hospital, Washington, DC; ^c^Plastic and Reconstructive Surgery, Department of Plastic Surgery, MedStar Georgetown University Hospital, Washington, DC; ^d^Private Practice Plastic Surgery, Chevy Chase, MD; ^e^Department of Plastic Surgery, Stanford University, Palo Alto, Calif; ^f^Department of Plastic Surgery, MD Anderson Cancer Center, Houston, Tex; ^g^Department of Plastic Surgery, Memorial Sloan-Kettering Cancer Center, New York, NY

**Keywords:** ADM, DermACELL, AlloDerm, NSM, ICG

## Abstract

**Background:** This article reports on the current use of acellular dermal matrix in breast reconstruction. **Methods:** A literature review of articles on acellular dermal matrix in breast reconstruction from January 1, 2010, through December 20, 2016, was performed and analyzed for trends in acellular dermal matrix use and differences between commonly used acellular dermal matrixes. **Findings:** Clinical findings varied but include improved cosmesis and more 1-stage reconstructions using acellular dermal matrix. Superiority of sterile versus aseptic acellular dermal matrixes was noted, and the increased incidence of red breast syndrome with AlloDerm was significant. The cost-effectiveness of acellular dermal matrix use despite increased upfront costs was also highlighted. Finally, the article emphasizes the importance of well-vascularized mastectomy flaps and the use of indocyanine green angiography as an adjunct in immediate reconstruction with acellular dermal matrix.

In the last decade, advancements in surgical technique and increased use of certain surgical devices and materials have fostered a transformation in postmastectomy breast reconstruction.^1^ A major innovation has been the use of acellular dermal matrix (ADM) to support prosthetic reconstructions. The main advantage of ADM use in this setting is greater control over the mastectomy space and the prosthetic device, which facilitates immediate 1- and 2-stage reconstructions.^1^ Accordingly, ADMs are now used in more than 60% of prosthetic-based reconstructions.^2^ At the same time, nipple-sparing mastectomy (NSM) has become the standard of care and accordingly the risk for necrosis has also increased, with up to a 40% rate of skin and nipple necrosis.^3^

This article reviews the latest clinical data describing the use of the most commonly used and available ADM products in postmastectomy breast reconstruction. This article attempts to highlight how available ADMs may differ and which materials and techniques are supported by current evidence.

## METHODS

A literature search was conducted in PubMed using the search terms “acellular dermal matrix,” “breast reconstruction,” and “mastectomy.” The initial search identified 382 items. To be included in the current article, articles were limited to human studies, published in English, from January 1, 2010, through December 20, 2016. The time period was selected to focus on more recent studies and minimize the influence of surgeon learning curve and early technical refinements. Case reports and small case series were excluded. Systematic reviews, meta-analysis, larger case series, retrospective studies, and prospective studies that evaluated the clinical use of ADM for breast reconstruction following mastectomy were included.

Included studies were evaluated for postoperative complication rates and categorized as ADM-only, ADM versus no-ADM, or ADM versus ADM. Rates of postoperative complications were also compared for aseptic and sterile ADM products.

## BACKGROUND

### Recent advances in mastectomy and reconstruction

As with the overall growth in mastectomies, the use of skin-sparing mastectomy and NSM has increased for breast cancer treatment and prophylaxis.^4^ Both allow for immediate reconstructions, including 1-stage (direct-to-implant or DTI) reconstructions. When insufficient skin remains to create a breast mound in 1 stage, a 2-stage approach using a tissue expander may be necessary. The 2-stage approach also allows vascularity of the mastectomy flaps to improve over time.

During prosthetic reconstructions, control and definition of the breast footplate and the implant or tissue expander are essential to optimize aesthetic outcomes. The introduction of ADM to breast reconstruction enhances surgeon control over the mastectomy space and facilitates 1- and 2-stage immediate reconstructions by strictly defining the implant pocket and off-loading the tension of the implant on the mastectomy skin envelope.

### Acellular dermal matrix

The expanding class of ADM comprises biological materials derived from human or animal (bovine, porcine) tissues. The source tissues are treated using proprietary, device-specific processes to remove cells and antigens and introduce varying degrees of collagen cross-linking. The resulting acellular materials contain intact extracellular matrix (collagen fibers, elastin, hyaluronic acid, fibronectin, proteoglycans), the specific composition of which varies across products. Available ADM are either prepared using aseptic techniques or terminally sterilized (Table 1).

A core feature of ADM is its capacity to support cell ingrowth, neovascularization, and integration into host tissues. This feature contrasts with synthetic products, which incite an inflammatory process that results in degradation of the device and replacement with scar. An ideal ADM provides durable pliability and strength while minimizing inflammatory response and fostering robust tissue ingrowth and integration.

#### ADM and postmastectomy breast reconstruction

The use of ADM for postmastectomy breast reconstruction was first reported by Breuing and Warren^5^ in 2005. In this technique, the ADM is sutured between the lower border of the pectoralis major muscle and the chest wall, creating a sling that reestablishes the lower pole of the breast. The ADM sling augments the inferior aspect of the subpectoral pocket and provides inferolateral implant coverage and support.^6^

The use of ADM may allow for greater initial tissue expander fill volumes or facilitate single-stage (DTI) procedures, obviating the need for tissue expanders.^6^^,^^7^ With skin-sparing techniques such as NSM, the ADM sling may also improve aesthetic outcomes in the lower pole, fostering a more natural-looking breast ptosis.^5^^,^^6^

Initial reports suggested a higher rate of certain postoperative complications associated with the use of ADM in breast reconstruction. However, the broad diversity of ADM products, patients with mastectomy, surgical techniques, and study methodologies contributes to wide variation in the outcomes of ADM studies, leaving surgeons with inconsistent guidance on postoperative risks and surgical best practices.^8^^-^10

#### Postoperative complications following breast reconstruction

Multiple risk factors are thought to influence the outcomes of breast reconstruction, including patient characteristics, cancer therapeutics, surgical factors, and qualities of the ADM used (Table 2).^11^^-^22

The baseline complication rate of 2.5% for implant reconstruction^23^ has increased with ADM use. In a recent meta-analysis of 23 studies, the relative risks (RRs) for major infection (RR = 2.74; 95% confidence interval [CI], 1.70-4.42), overall infection (RR = 1.42; 95% CI, 1.02-1.99), seroma (RR = 1.41; 95% CI, 1.12-1.78), and flap necrosis (RR = 1.44; 95% CI, 1.11-1.87) were significantly higher with ADM.^24^ In addition, ADM was associated with reduced risks for capsular contracture and implant malposition.^24^

## RESULTS

The results of our literature review are presented in Tables 3-6. Studies are grouped by comparator groups: ADM versus no-ADM (Table 3), ADM-only (no comparator; Table 4), ADM versus ADM (Table 5), and aseptic versus sterile ADM (Table 6).

Included studies comparing the use of ADM with submuscular prosthetic coverage without ADM (Table 3) reflect inconsistent outcomes.^15^^,^^25^^-^35 Statistically significant differences between the ADM and non-ADM groups were reported by several studies, with higher rates of total complications, infection, and seroma in the ADM groups. One study also reported a significantly higher rate of flap necrosis with ADM.^33^ Several studies reported significantly higher rates of explantation with ADM. Importantly, many studies found no significant differences in complications between groups, and some reported higher rates of explantation and unplanned return to the operating room in the non-ADM groups.

More recent studies evaluating the use of different ADMs are illustrated in Tables 4 and 5. Single-group studies (Table 4) reported outcomes with a range of ADM types.^7^^,^^9^^,^^36^^-^43 There were 4 prospective and 7 retrospective studies; these included 3 multicenter and 8 single-center studies. With the exception of one study, the complication rates were relatively modest and within previously reported ranges (total complications: 3.9%-16.4%). The exception was a study from the Netherlands of Strattice (porcine ADM) use in 88 patients, which reported an extremely high complication rate (78%) and reoperation in 22.7% of cases. 37 This study also noted a high rate of red breast syndrome (RBS), which occurred in 14.5% of cases. The largest study (863 women, 1584 breasts), based predominantly on AlloDerm use (93% of cases), reported very low rates of seroma (1.1%) and capsular contracture (0.8%).^38^ One study compared different material thicknesses (AlloDerm) and reported a trend toward higher complication rates with thicker ADM.^36^

The question of which ADM supports the best outcomes was addressed by studies comparing 2 or more ADMs (Table 5).^16^^-^18^,^^21^^,^^44^^-^55 There were 15 retrospective studies and 1 randomized study,^48^ and all study data were derived from single centers or individual surgeons. Rates of total complications ranged widely, from 8.6% to 47.7%. The one randomized trial found no significant differences between groups in total or individual complications.^48^ Several studies reported significant differences between ADM in total or specific complications. However, there was no consistency in these findings between studies. Interesting significant findings included a much lower rate of RBS (0% vs 26%; *P* = .0001) and fewer days to drain removal (15.8 vs 20.6; *P* = .017) with DermACELL compared with AlloDerm RTU (ready to use)^46^; a significantly lower rate of seroma with fenestrated versus nonfenestrated ADM (11.1% vs 20%; *P* = .0098)^17^; and a significantly higher rate of seroma with AlloDerm versus Strattice (12.7% vs 1.4%; *P* = .0003).^52^

Finally, studies comparing aseptic and sterile preparations of ADM are compared in Table 6.^56^^-^61 All were single-center studies, all but one were retrospective, and all compared aseptic (or freeze-dried) and sterile (or RTU) AlloDerm. Overall, the studies suggest a trend toward higher rates of complications, particularly infection, with aseptic ADM. Two studies reported significantly higher complication rates with aseptic ADM than with sterile ADM (total complications: 41.9% vs 27%, *P* = .046; infection: 20% vs 8.5%, *P* = .0088).^58^^,^^61^ The outlier was a small retrospective study that reported extremely high rates of seroma with AlloDerm RTU compared with aseptic AlloDerm or no ADM (66.6% vs 8% vs 8.3%, respectively; *P* = .003).^60^

## DISCUSSION

The introduction of ADM has revolutionized postmastectomy breast reconstruction by enabling 1- and 2-stage reconstructions, improving surgeon control over the implant and mastectomy space, and fostering improved aesthetic outcomes. Considered together, the studies included in this review demonstrate differing levels of success with all of the available ADMs. Current data do not yet identify any one optimal ADM. However, the evidence does suggest certain trends: ADM appears to be associated with an increased risk for infection and seroma and a decreased risk for capsular contracture.

### Differences in ADM characteristics

Important differences between ADM products include tissue source (human, bovine, porcine), methods of decellularization and antigen removal, use of supplemental cross-linking, and final preparation (aseptic vs sterile). These characteristics may influence how efficiently the ADM integrates into the host tissues, its surgical utility and durability, and tolerance by the host. Rapid host acceptance of the ADM, with minimal inflammation and an organized host response of cell infiltration into the ADM, will optimize outcomes. Differences between ADM may influence the rate and extent of these processes, possibly affecting the local inflammatory response and risks for infection and seroma formation.^62^

Suboptimal decellularization may leave cell remnants that can induce an inflammatory response when implanted. Conversely, excessive damage to the extracellular matrix during processing (chemical cross-linking, radiation, etc) may also increase inflammation while reducing cellular and vascular infiltration of the material, limiting integration.^63^^-^66 The impact of these differences is illustrated by preclinical studies that evaluate components of host response on implantation of the ADM. For example, a study using a rat model demonstrated differing modes and degrees of cellular ingrowth in different ADM, with the highest degree of cell ingrowth with DermACELL and lowest with AlloDerm.^67^

### Aseptic versus sterile ADM

Another key difference between ADM products is their preparation as aseptic or sterile final products (see Table 1). Aseptic materials (such as FlexHD or the older form of AlloDerm) are prepared using aseptic handling throughout manufacture. Sterile ADMs are exposed to γ-radiation or other forms of terminal sterilization following manufacture and packaging. By definition, aseptic products have a sterility assurance level (SAL) of 10^−3^; stated another way, a chance of infection from the product itself is one in a thousand (eg, AlloDerm RTU, FlexHD). Sterile products, the standard for implantable medical devices such as breast implants, have an SAL of 10^−6^. Therefore, the chance of an infection from a device that is sterile is one in a million (eg, DermACELL, AlloMax [ie, NeoForm]).

It has been proposed that sterile ADM may be associated with lower rates of infection than with aseptic ADM following breast reconstruction. Current evidence (Table 6) clearly supports a trend toward lower infection rates with sterile ADM. One prospective study by Weichman et al^61^ reported a significantly lower rate of infections with sterile versus aseptic ADM (8.5% vs 20%; *P* = .0088). Lewis et al^58^ reported a significantly lower rate of total complications with sterile versus aseptic ADM (27% vs 41.9%; *P* = .046); no significant differences were found for individual complications. An older retrospective study of 31 reconstructions using NeoForm (a sterile ADM now called AlloMax) reported no cases of infection, seroma, erythema, or foreign body reaction.^68^ A more recent study of 65 reconstructions in 39 patients using sterile ADM reported an overall complication rate of 4.6% (3 breasts), which included 1 case of cellulitis (1.5%) and 2 cases of mastectomy flap necrosis (3%).^42^ Similarly, studies comparing sterile ADM, such as AlloDerm RTU and DermACELL (see Table 5), have reported low rates of infection (0%-6%) with each ADM.^16^^,^^46^

The majority of older studies of ADM in breast reconstruction utilized aseptic AlloDerm, which was replaced by sterile AlloDerm RTU in 2011. The majority of studies reporting the use of AlloDerm evaluated clinical use prior to 2011 and therefore reflect the use of aseptic ADM. These studies, although numerous, are less relevant for current products, as they demonstrate infection rates that are higher than would be expected with sterile ADM.

### Patient selection for use of ADM

Overall, studies suggest that ADM may be less effective in certain patients, such as the morbidly obese (body mass index >40 kg/m^2^), those with prior mastectomy and radiation therapy, those with severe vascular compromise to the skin flaps immediately following mastectomy, and those who are active users of tobacco products.^1^ Some authors suggest that ADMs are less effective in patients with delayed reconstruction, exposure to radiation, a history of smoking, poor skin flap perfusion, or morbidly obesity.^1^^,^^13^^,^^69^

#### ADM and radiotherapy

The use of ADM in the context of radiotherapy remains controversial. Some studies have reported no difference in risk for complications when implanted ADM is exposed to radiation, whereas others have found higher complication rates in irradiated versus nonirradiated breasts with ADM.^7^^,^^70^^,^^71^ Moyer et al^72^ evaluated 27 patients who underwent bilateral reconstruction with ADM and subsequent unilateral radiotherapy. Capsular contracture occurred in 9 patients (33%) and was limited to the irradiated side; 75% of all other complications also occurred in the irradiated side. The authors concluded that ADM limited the elastosis and chronic inflammation normally seen in irradiated implant-based reconstructions, which may mitigate capsular contracture, encouraging the use of ADM in these cases.

### Red breast syndrome

The clinical signs of RBS overlap to some degree with cellulitis and infection (eg, erythema, swelling, warmth) but generally do not include fever or laboratory abnormalities.^73^ The condition is thought to be a type of delayed hypersensitivity reaction.^74^ Potential contributors to RBS include characteristics of the ADM and additives used in the packaging of some ADM.^58^ Several studies reported a high incidence of RBS with certain ADMs and lower rates in others.^46^^,^^58^ In one study, the rate of RBS was 0% with DermACELL and 26% with AlloDerm RTU (*P* = .0001), suggesting that not all ADMs are equal and the host response to each may vary.^46^

### Optimizing mastectomy flap quality: The use of laser-assisted indocyanine green angiography

Many now believe that it is the vascularity of the mastectomy flaps that most dictates complications.^13^ Poor vascularity may occur in excessively thin, traumatized, or widely undermined flaps.^75^ It follows that the recellularization and integration of any ADM will depend on adequate vascularity in the recipient area.

Laser-assisted indocyanine green angiography (LA-ICGA) is a vascular imaging methodology that can be used in the intraoperative or postoperative setting to visually assess blood flow within the mastectomy skin envelope. ICG angiography provides real-time assessment of tissue perfusion that has been correlated with clinical outcomes^76^^-^80 and guides surgical decision-making, such as intraoperative tissue resection and staging of reconstruction procedures. 81^,^^82^ The SPY Elite system (Novadaq, Mississauga, Ontario, Canada), an advanced LA-ICGA device, assigns numeric values to levels of perfusion detected through ICG fluorescence. In one study, an SPY value of 7 or less accurately predicted flap necrosis, with 88% sensitivity and 83% specificity.^83^

Clinical studies of the use of LA-ICGA in postmastectomy breast reconstruction have demonstrated a high degree of correlation between intraoperative perfusion values on LA-ICG (SPY) and postoperative skin flap outcomes.^11^^,^^12^^,^^84^ Two recent retrospective studies from the Mayo Clinic further support the utility of LA-ICGA for the identification of tissue at risk for complications. The first reviewed 467 consecutive reconstructions that spanned the introduction of LA-ICGA at the center (254 without and 213 with LA-ICGA).^85^ The rates of total complications without SPY and with SPY use were 13.8% versus 6.6% (*P* = .01). Flap necrosis decreased significantly after LA-ICGA became available (6.7% vs 0.9%; *P* = .02). At the same time, the use of single-stage (DTI) reconstructions increased significantly (from 12% to 32%; *P* < .001) due to confidence with the vascular status of the mastectomy flaps. The second, larger analysis reviewed 942 reconstructions (590 without and 352 with LA-ICGA).^86^ Again, total complications (17% vs 6%; *P* < .001) and skin flap necrosis (6% vs 1%; *P* < .001) decreased significantly with the use of LA-ICGA. Importantly, the use of single-stage (DTI) procedures increased significantly (from 7% to 23%; *P* < .001) and NSMs were performed more frequently (28%-32%; *P* = .21).

### Economic costs and benefits

The use of ADM both increases material costs and potentially reduces the need for subsequent procedures. One retrospective analysis of 367 patients undergoing prosthetic reconstructions found that average initial costs were higher when ADM was used ($6868 with ADM vs $5615 without ADM), but average total costs over 2 years were lower ($11,862 vs $12,319).^87^ This shift was driven by significantly lower costs for postreconstructive events in the absence of ADM ($5176 with ADM vs $6704 without ADM; *P* < .05).

The type of reconstruction also influences costs.^88^^-^90 A study comparing Medicare reimbursements costs for tissue expander reconstructions, with or with ADM, and single-stage procedures with ADM found that costs were highest for tissue expander plus ADM ($11,255.78), followed by tissue expander without ADM ($10,934.18), and then single-stage with ADM ($5432.02).^89^ When the estimated costs of complications (based on published literature) were included, the costs of tissue expander reconstructions were similar ($11,829.02 with ADM vs $11,238.60 without ADM), but single-stage ADM reconstructions remained highly cost-effective ($5909.83).

## CONCLUSIONS

Increasing numbers of women are demanding techniques that improve aesthetic outcomes following reconstruction, such as skin- and nipple-sparing procedures, that can be accomplished safely in the immediate setting. The use of ADM has revolutionized the approach to immediate breast reconstruction by providing surgeons with greater control and flexibility in performing the procedure.^91^ Although current evidence suggests that the use of ADM may increase risk for certain complications (infection, seroma), appropriate patient selection and well-vascularized flaps can minimize or eliminate these risks.

Certain features of ADM have been promoted by surgeons for use in breast reconstruction, including a general preference for sterile ADM, which may be associated with a lower risk for infection; human ADM, because of the greater elasticity of human skin; and thinner sheets of ADM, which may facilitate integration into host tissues.^13^ As ADM-based reconstruction becomes the standard of care and the performance of specific ADMs is shown to be more and more similar, the overriding factor in ADM choice will be economic.

## Figures and Tables

**Table 1:** ADM commonly used for breast reconstruction*

ADM	Source	Aseptic/sterile
AlloDerm (LifeCell Corp, Branchburg, NJ)	Human	Aseptic
AlloDerm RTU (LifeCell Corp, Branchburg, NJ)	Human	Sterile (SAL 10^−3^)
AlloMax (Davol Inc, Murray Hill, NJ)	Human	Sterile (SAL 10^−6^)
FlexHD (Ethicon Inc, Somerville, NJ)	Human	Aseptic
DermaMatrix (MTF/Synthes CMF, West Chester, Pa)	Human	Sterile (SAL 10^−6^)
DermACELL (LifeNet Health, Virginia Beach, Va)	Human	Sterile (SAL 10^−6^)
NeoForm (Mentor, Santa Barbara, Calif)	Human	Sterile (SAL 10^−6^)
Strattice (LifeCell Corp, Branchburg, NJ)	Porcine	Sterile (SAL 10^−3^)
Permacol (Covidien, Boulder, Colo)	Porcine	Sterile (SAL 10^−6^)
SurgiMend PRS (TEI Biosciences Inc, Boston, Mass)	Bovine	Sterile (SAL 10^−6^)

*ADM indicates acellular dermal matrix; RTU, ready to use; and SAL, sterility assurance level.

**Table 2:** Factors associated with increased risk for complications following postmastectomy breast reconstruction*

Category	Risk factor
Patient characteristics	Age >50 y
	Smoking history
	BMI >30 kg/m^2^
	Diabetes mellitus
	Larger breast size
Medical factors	Postoperative chemotherapy
	Postoperative radiotherapy
	History of radiotherapy
	Current steroid use
Surgical factors	Greater expander fill volume
	Axillary dissection
	Longer operative time
	Nipple-sparing mastectomy
	Poor-quality mastectomy flap
	Insufficient vascularity
	Thin flaps
	Extensive undermining of flaps
ADM characteristics	Aseptic vs sterile
	Perforated vs intact
	Contoured vs flat
	Greater ADM surface area

*From references 11-13, 43-51. ADM indicates acellular dermal matrix; BMI, body mass index.

**Table 3:** Studies comparing reconstructions with the use of ADM to no-ADM*

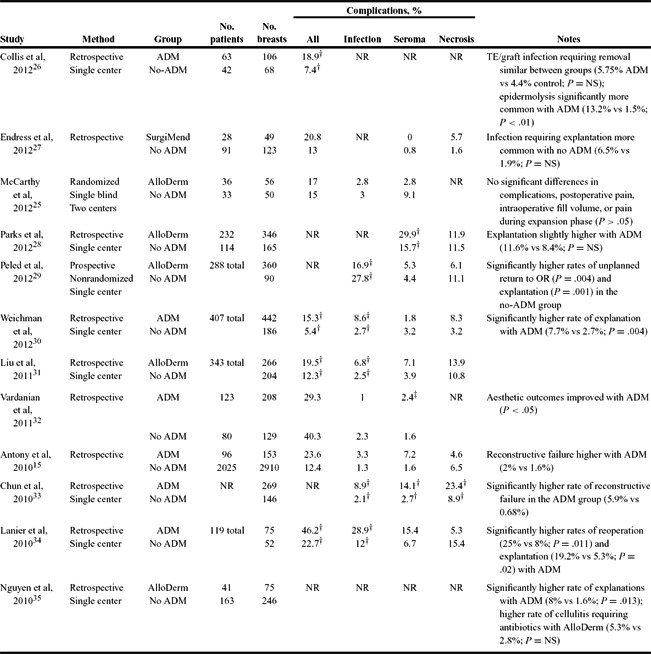

*ADM indicates aceullar dermal matrix; NR, not reported; TE, tissue expander; NS, nonsignificant; and OR, operating room.

^†^Statistically significant difference (*P* < .05).

^‡^Seroma/hematoma combined.

**Table 4:** Studies reporting postoperative complications with the use of ADM (no-comparator group)*

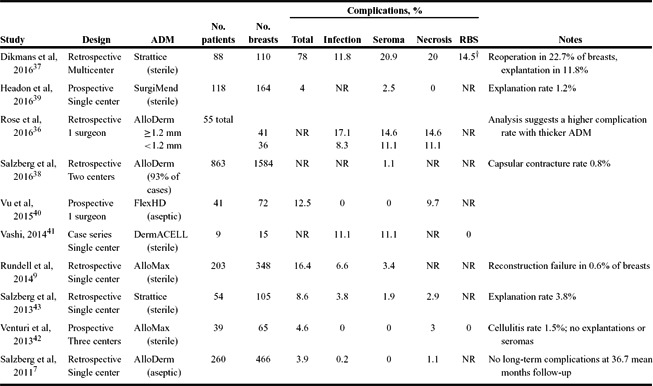

*ADM indicates acellular dermal matrix; RBS, red breast syndrome; and NR, not reported.

^†^Reported as erythema/inflammation.

**Table 5:** Studies comparing different ADM in postmastectomy breast reconstruction*

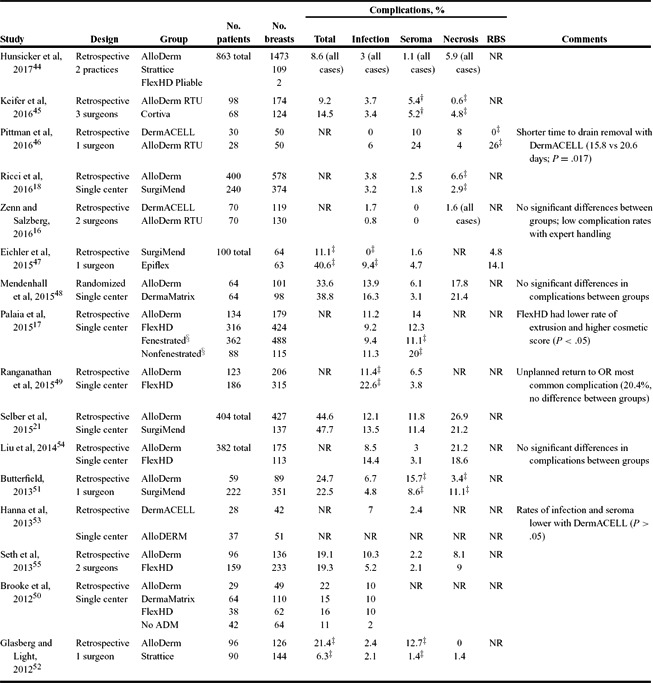

*ADM indicates aceullar dermal matrix; RBS, red breast syndrome; NR, not reported; RTU, ready to use; OR, operating room.

^†^Seroma/hematoma combined.

^‡^Statistically different between groups (*P* < .05).

^§^AlloDerm and FlexHD combined.

**Table 6:** Outcomes of studies comparing the use of aseptic ADM to sterile ADM*

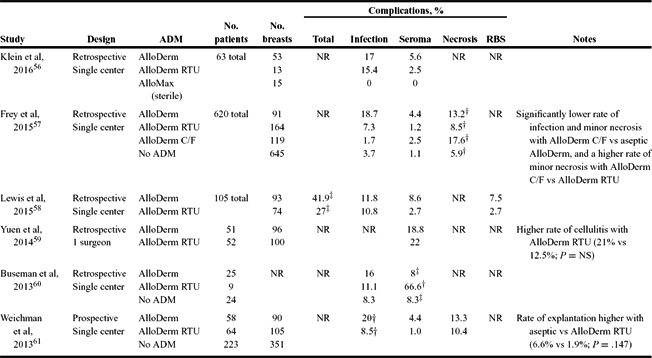

*ADM indicates acellular dermal matrix; RBS, red breast syndrome; NR, not reported; RTU, ready-to-use (sterile); and C/F, contour-fenestrated.

^†^Combined major and minor flap necrosis rates.

^‡^Statistically significant difference (*P* < .05).
